# Supplementary material for: Parental Age Effects on Offspring Telomere Length Across Vertebrates: A Meta‐Analysis
Source: Mol Ecol. 2026 Jan 20;35(2):e70215. doi: 10.1111/mec.70215 (PMC12817657; doi:10.1111/mec.70215)
Supplement: Supplementary file 1 — Figure S1: Non‐aggregated forest plot of the individual effect size estimates included in the meta‐analysis of parental age at conception effects on offspring telomere length. Figure S2:. Bar plots showing the distribution of estimates of parental age effect on offspring telomere lengths across (a) parental sex, (b) offspring sex, (c) sperm production seasonality, (d) cell type from which the telomeres were extracted, (e) offspring age at measurement, (f) laboratory method, (g) whether within−/between‐parent effects were accounted for, (h) whether parental environment effects were controlled for, and (i) whether the parent's identity was controlled for in humans. Figure S3:. Bar plots showing the distribution of estimates of parental age effect on offspring telomere lengths across (a) parental sex, (b) offspring sex (no study on female offspring was present), (c) sperm production seasonality, (d) cell type from which the telomeres were extracted, (e) offspring age at measurement, (f) laboratory method, (g) whether within−/between‐parent effects were accounted for, (h) whether parental environment effects were controlled for, and (i) whether the parent's identity was controlled for in non‐human vertebrates. Table S1:. Moderator estimates from the meta‐regression models of parental age at conception effect on offspring telomere length in humans (model 3.1) and 12 non‐human vertebrates (model 3.2), accounting for study, estimate, species and phylogenetic non‐independence and including both interactions (parental and offspring sex, offspring age and telomere tissue). Parentheses in the fixed effects subheadings indicate the reference level; p‐values < 0.05 are in bold and < 0.1 in italics. Table S2:. Post hoc test estimates and standard errors (SE) from models of parental age at conception effects on offspring telomere length for the laboratory method moderator. Table S3:. Post hoc test results for contrasts between different parental and offspring sex combinations, ext [file MEC-35-e70215-s001.docx]

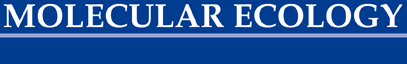


**Supplemental Information for:**

**Parental age effects on offspring telomere length across vertebrates: a meta-analysis**

Mariia Vlasova^1^*, Yuheng Sun^1^*, Heung Ying Janet Chik^1,2^, Hannah L. Dugdale^1^^


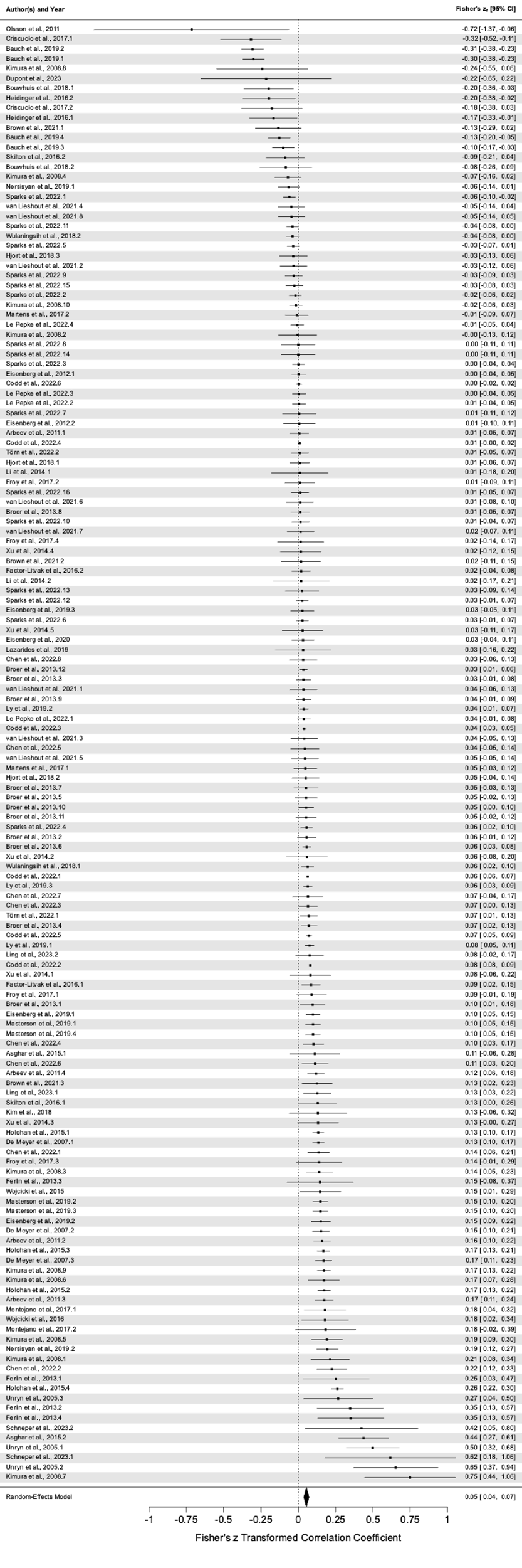


**Figure S1**. Non-aggregated forest plot of the individual effect size estimates included in the meta-analysis of parental age at conception effects on offspring telomere length. Each dot represents the estimate effect, with horizontal lines indicating the respective 95% confidence intervals and the dotted vertical line indicating zero.


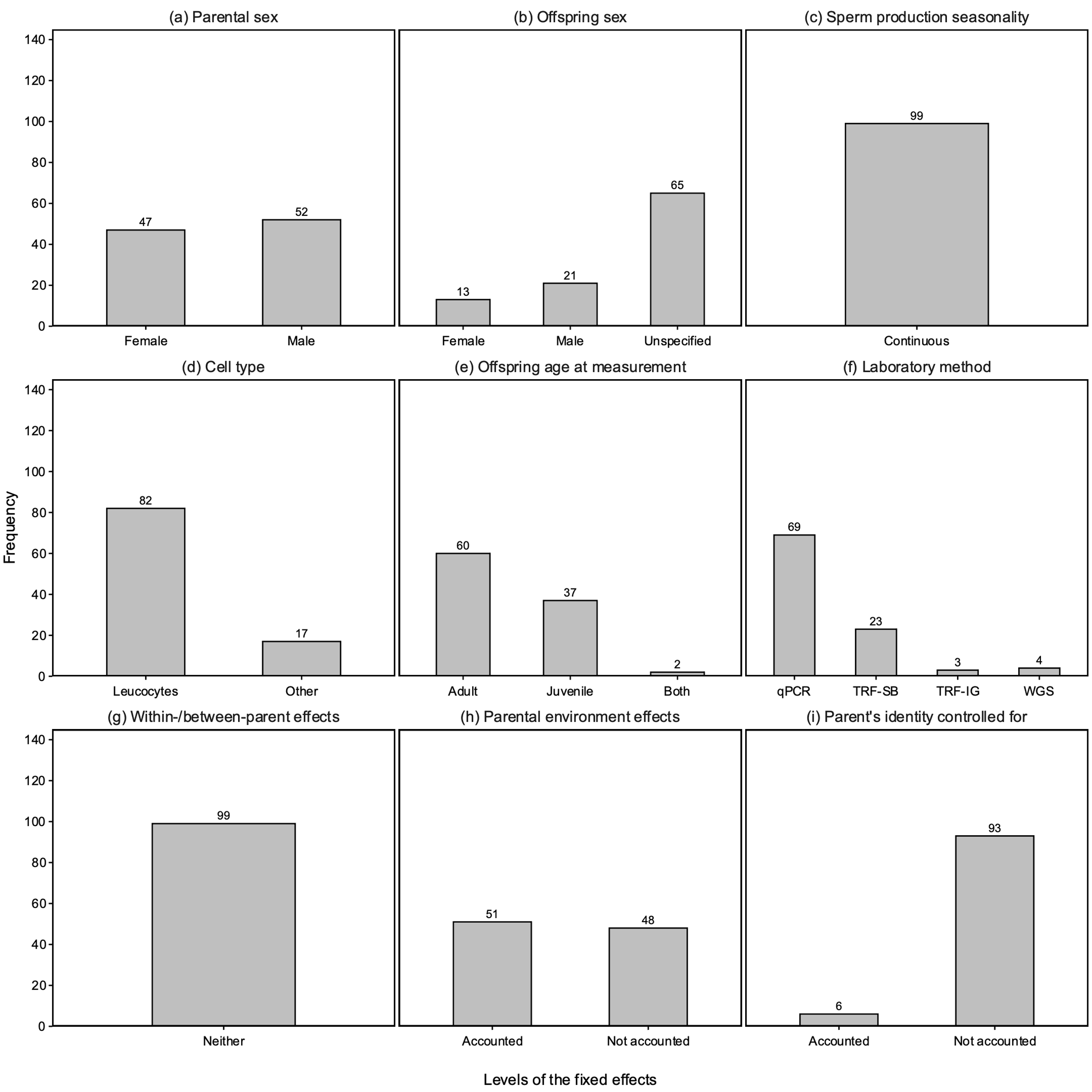


**Figure S2.** Bar plots showing the distribution of estimates of parental age effect on offspring telomere lengths across (a) parental sex, (b) offspring sex, (c) sperm production seasonality, (d) cell type from which the telomeres were extracted, (e) offspring age at measurement, (f) laboratory method, (g) whether within-/between-parent effects were accounted for, (h) whether parental environment effects were controlled for, and (i) whether the parent’s identity was controlled for in humans. Numbers above the bars represent sample sizes of estimates (N = 99).


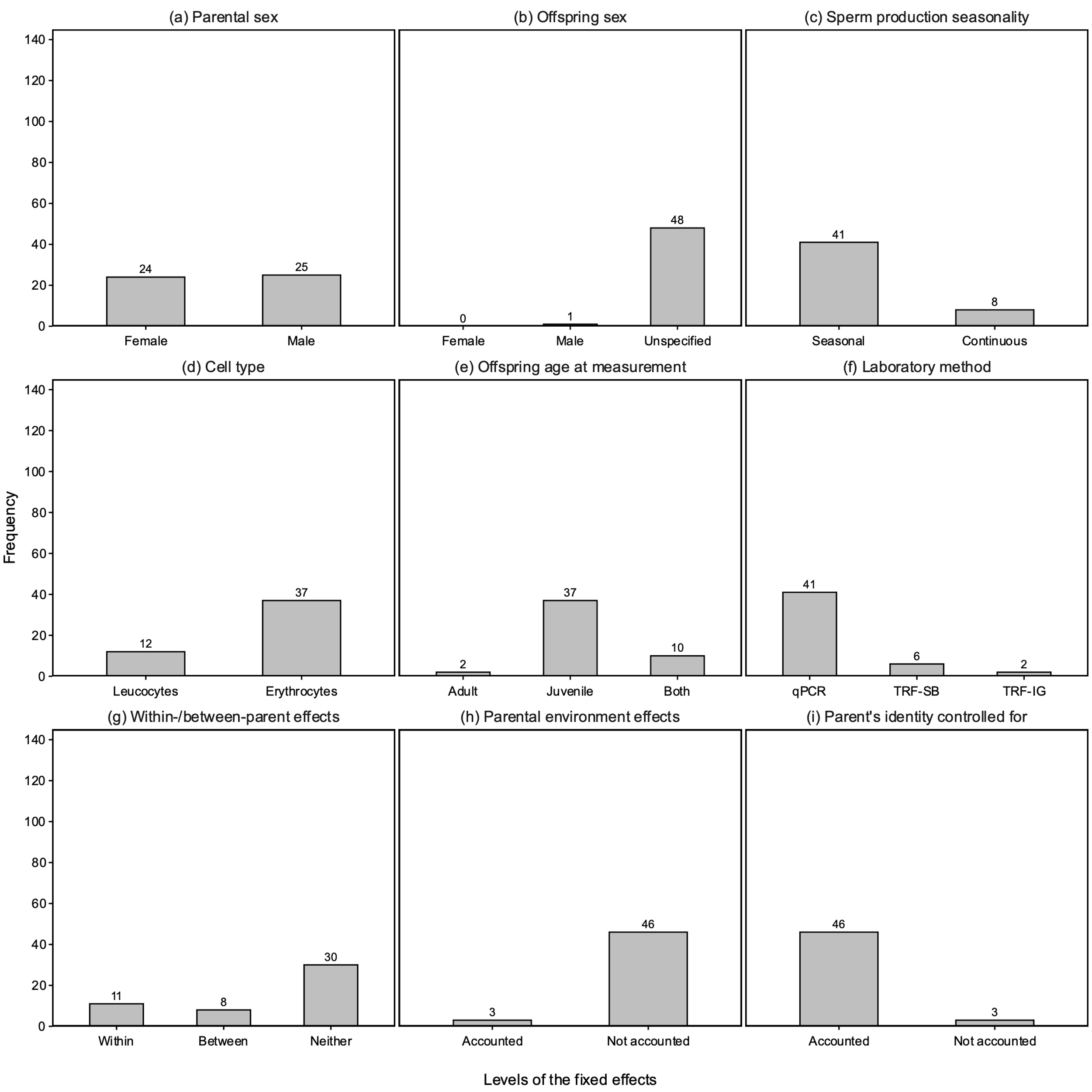


**Figure S3.** Bar plots showing the distribution of estimates of parental age effect on offspring telomere lengths across (a) parental sex, (b) offspring sex (no study on female offspring was present), (c) sperm production seasonality, (d) cell type from which the telomeres were extracted, (e) offspring age at measurement, (f) laboratory method, (g) whether within-/between-parent effects were accounted for, (h) whether parental environment effects were controlled for, and (i) whether the parent’s identity was controlled for in non-human vertebrates. Numbers above the bars represent sample sizes of estimates (N = 49).

**Table S1**. Moderator estimates from the meta-regression models of parental age at conception effect on offspring telomere length in humans (model 3.1) and 12 non-human vertebrates (model 3.2), accounting for study, estimate, species and phylogenetic non-independence and including both interactions (parental and offspring sex, offspring age and telomere tissue). Parentheses in the fixed effects subheadings indicate the reference level; p-values <0.05 are in bold and <0.1 in italics. SE = standard error, 95% CI = confidence interval, DF = degrees of freedom. TRF = terminal restriction fragments, WGS = whole genome sequencing.

|  | **Model 3.1 -  human subset** | | | **Model 3.2 - non-human vertebrate subset** | | |
| --- | --- | --- | --- | --- | --- | --- |
| **Fixed effects** | **estimate (SE)** | **95% CI** | **p-val** | **estimate (SE)** | **95% CI** | **p-val** |
| **intercept** | 0.032 (0.050) | [-0.066, 0.131] | 0.52 | -0.727 (0.360) | [-1.433, -0.022] | **0.04** |
| **Parental sex (Mother)** | | | | | | |
| **Father** | 0.129 (0.031) | [0.068, 0.190] | **< 0.001** | -0.015 (0.014) | [-0.042, 0.012] | 0.28 |
| **Offspring sex (Daughter)** | | | | | | |
| **Son** | 0.114 (0.034) | [0.048, 0.180] | **< 0.001** |  |  |  |
| **Unspecified-sex offspring** | 0.087 (0.030) | [0.028, 0.145] | **0.004** | 0.670 (0.353) | [-0.021, 1.362] | *0.06* |
| **Telomere tissue (other)** | | | | | | |
| **Erythrocytes** |  |  |  | -0.0522 (0.0725) | [-0.1943, 0.0898] | 0.47 |
| **Leukocytes** | -0.064 (0.048) | [-0.157, 0.029] | 0.18 |  |  |  |
| **Sperm production seasonality (Continuous)** | | | | | | |
| **Seasonal** |  |  |  | 0.059 (0.083) | [-0.104, 0.221] | 0.48 |
| **Parental environment effects controlled for (No)** | | | | | | |
| **Present** | 0.003 (0.018) | [-0.032, 0.039] | 0.85 | -0.027 (0.083) | [-0.189, 0.135] | 0.74 |
| **Parent’s identity controlled for (No)** | | | | | | |
| **Yes** | -0.023 (0.028) | [-0.078, 0.032] | 0.42 | 0.229 (0.090) | [0.053, 0.405] | **0.011** |
| **Within-/ between-parent effects (Neither)** | | | | | | |
| **Between** |  |  |  | 0.021 (0.024) | [-0.027, 0.069] | 0.40 |
| **Within** |  |  |  | -0.025 (0.024) | [-0.073, 0.023] | 0.30 |
| **Offspring age at measurement (adult)** | | | | | | |
| **Both** | -0.003 (0.039) | [-0.079, 0.074] | 0.94 | -0.167 (0.089) | [-0.342, 0.008] | *0.06* |
| **Juvenile** | -0.045 (0.053) | [-0.149, 0.060] | 0.40 | -0.158 (0.081) | [-0.315, 0.000] | *0.05* |
| **Laboratory method (qPCR)** | | | | | | |
| **TRF in-gel** | 0.412 (0.073) | [0.268, 0.556] | **< 0.001** | -0.157 (0.088) | [-0.328, 0.015] | *0.07* |
| **TRF Southern blot** | 0.061 (0.019) | [0.023, 0.099] | **0.002** | -0.209 (0.063) | [-0.332, -0.085] | **0.001** |
| **WGS** | 0.0092 (0.0334) | [-0.0563, 0.0747] | 0.78 |  |  |  |
| **Interaction parental sex:offspring sex (mother : daughter)** | | | | | | |
| **Father : Son** | -0.131 (0.043) | [-0.215, -0.047] | **0.002** |  |  |  |
| **Father : unspecified-sex offspring** | -0.104 (0.034) | [-0.170, -0.038] | **0.002** |  |  |  |
| **Interaction telomere tissue : offspring age (other : adult)** | | | | | | |
| **Erythrocytes : Both juvenile and adult** |  |  |  | 0.010 (0.049) | [-0.086, 0.105] | 0.84 |
| **Erythrocytes : Juvenile** |  |  |  |  |  |  |
| **Leucocytes : Juvenile** | 0.018 (0.054) | [-0.087, 0.124] | 0.74 |  |  |  |
| **Random effects** | **Estimate (σ^2^)** | **95% CI** | **Proportion heterogeneity explained** | **Estimate (σ^2^)** | **95% CI** | **Proportion heterogeneity explained** |
| **Study ID** | 0.000 | 0.000, 0.002 | 0.191 | 0.003 | 0.000, 0.018 | 0.767 |
| **Estimate ID** | 0.001 | 0.001, 0.003 | 0.750 | 0.001 | 0.000, 0.003 | 0.200 |
| **Phylogeny** |  |  |  | 0 | 0.000, 0.001 | < 0.001 |
| **I^2^ total** | 0.941 | | | 0.967 | | |
| **I^2^ residual** | 0.059 | | | 0.033 | | |
| **Observations (N)** | 99 | | | 49 | | |
| **AIC** | -202.97 | | | -87.20 | | |
| **BIC** | -158.85 | | | -55.04 | | |
| **Log likelihood** | 118.48 | | | 60.60 | | |
| **Deviance** | 226.71 | | | 89.70 | | |
| **DF residual** | 84 | | | 35 | | |

**Table S2.** Post-hoc test estimates and standard errors (SE) from models of parental age at conception effects on offspring telomere length for the laboratory method moderator. TRF = terminal restriction fragments, WGS = whole genome sequencing.

|  | **Human studies** | | **Non-human studies** | |
| --- | --- | --- | --- | --- |
| **Laboratory method** | **estimate** | **SE** | **estimate** | **SE** |
| qPCR | 0.067 | 0.021 | -0.403 | 0.186 |
| TRF in-gel | 0.478 | 0.076 | -0.558 | 0.208 |
| TRF Southern blot | 0.126 | 0.027 | -0.613 | 0.189 |
| WGS | 0.077 | 0.040 |  |  |

**Table S3.** Post-hoc test results for contrasts between different parental and offspring sex combinations, extracted from the meta-regression model of parental age at conception effects on offspring telomere length in human studies. “F F - M F” represents contrasting the female-parent-female-offspring combination with the male-parent-female-offspring combination; the same applies to the other contrasts, with F = female, M = male, and U = unspecified-sex. SE = standard error. Significant contrasts are in bold.

| **contrast** | **estimate** | **SE** | **z ratio** | **p value** |
| --- | --- | --- | --- | --- |
| **F F - M F** | **-0.128** | **0.031** | **-4.114** | **<0.001** |
| **F F - F U** | **-0.085** | **0.030** | **-2.871** | **0.047** |
| **F F - M U** | **-0.110** | **0.029** | **-3.840** | **0.002** |
| **F F - F M** | **-0.116** | **0.033** | **-3.480** | **0.007** |
| **F F - M M** | **-0.112** | **0.032** | **-3.523** | **0.006** |
| M F - F U | 0.043 | 0.026 | 1.639 | 0.57 |
| M F - M U | 0.018 | 0.024 | 0.751 | 0.98 |
| M F - F M | 0.013 | 0.031 | 0.409 | 0.99 |
| M F - M M | 0.016 | 0.028 | 0.567 | 0.99 |
| F U - M U | -0.024 | 0.013 | -1.825 | 0.45 |
| F U - F M | -0.030 | 0.028 | -1.063 | 0.90 |
| F U - M M | -0.027 | 0.026 | -1.036 | 0.91 |
| M U - F M | -0.006 | 0.027 | -0.208 | 1.00 |
| M U - M M | -0.002 | 0.024 | -0.094 | 1.00 |
| F M - M M | 0.003 | 0.031 | 0.110 | 1.00 |
